# Supplementary material for: Physico-Chemical Properties of Sugar Beet Pectin-Sodium Caseinate Conjugates via Different Interaction Mechanisms
Source: Foods. 2019 Jun 3;8(6):192. doi: 10.3390/foods8060192 (PMC6617378; doi:10.3390/foods8060192)
Supplement: Supplementary file 1 [file foods-08-00192-s001.pdf]

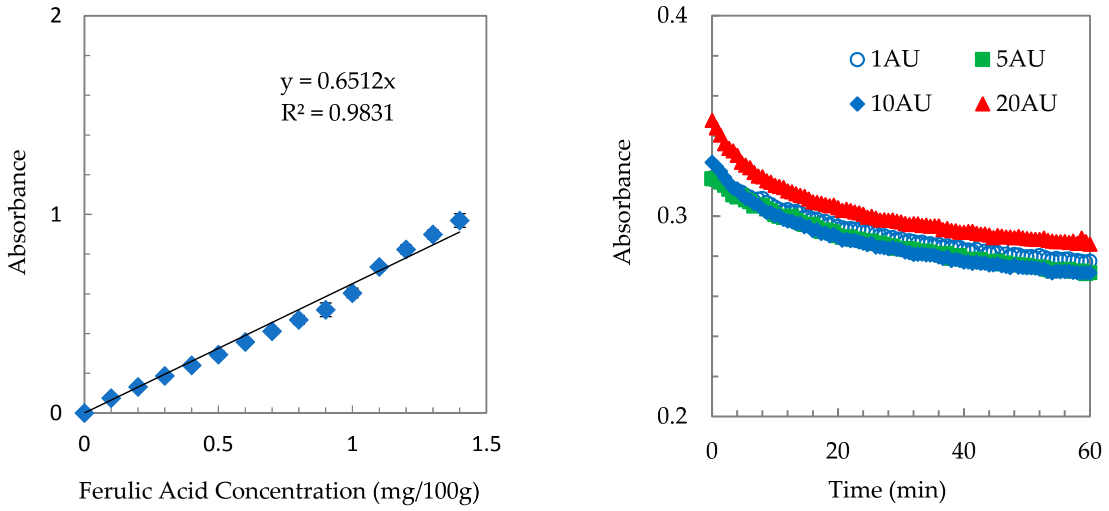

(a) (b)

Figure S1. (a) Standard absorbance of ferulic acid concentration in sugar beet pectin at 325 nm wavelength and 25 ℃; (b) Influence of different enzyme concentrations on time-dependence of absorbance at 325 nm of 0.4 w/w % sugar beet pectin dispersions at pH 5 (citrate buffer 50mM) at 25 °C. Error bars correspond to ±1 standard deviation of triplicate freshly prepared samples, and each sample was measured three times.

Table S1. Effect of thermal treatment at 80 °C for 10 min on particle size and ζ-potential of SBP-SC conjugates at pH 4.5 and pH 7 at 20 °C. The different letters (a, b, c, d, e, f, g, h, I, and j) represent significant differences among samples (*p*-value < 0.05).

**SBP:SC**	**z-Average Radius (nm)**	**ζ-Potential (mV)**
1:1 P pH4.5	259 ± 23 ^{a}	-34.04 ± 0.67 ^{e}
1:1 P pH 4.5 heat 80°C	223± 11^{b}	-33.55 ± 0.66 ^{e}
1:1 E pH4.5	188 ± 18 ^{c}	-29.18 ± 0.74 ^{f}
1:1 E pH 4.5 heat 80°C	187 ± 9 ^{c}	-31.81 ± 0.31 ^{g}
1:1 M pH4.5	>2000	-29.18 ± 0.52 ^{f}
1:1 M pH 4.5 heat 80°C	> 2000	-28.26 ± 0.24 ^{h}
1:1 P pH7	183 ± 31^{c}	-36.12 ± 0.63 ^{I}
1:1 P pH 7 heat 80°C	147 ± 4 ^{d}	-38.79 ± 1.60 ^{j}
1:1 E pH7	188 ± 19 ^{c}	-36.04 ± 0.35 ^{I}
1:1 E pH 7 heat 80°C	166 ± 16 ^{c}	-35.26 ± 0.31 ^{I}
1:1 M pH7	213 ± 19 ^{b}	-36.64 ± 0.54 ^{I}
1:1 M pH 7 heat 80°C	160 ± 14 ^{c}	-36.34 ± 0.23 ^{I}
